# *Lactobacillus plantarum* BSGP201683 Isolated from Giant Panda Feces Attenuated Inflammation and Improved Gut Microflora in Mice Challenged with Enterotoxigenic *Escherichia coli*

**DOI:** 10.3389/fmicb.2017.01885

**Published:** 2017-09-26

**Authors:** Qian Liu, Xueqin Ni, Qiang Wang, Zhirong Peng, Lili Niu, Hengsong Wang, Yi Zhou, Hao Sun, Kangcheng Pan, Bo Jing, Dong Zeng

**Affiliations:** ^1^Animal Microecology Institute, College of Veterinary Medicine, Sichuan Agricultural University, Chengdu, China; ^2^Chengdu Wildlife Institute, Chengdu Zoo, Chengdu, China; ^3^Key Laboratory of Animal Disease and Human Health of Sichuan Province, Chengdu, China

**Keywords:** *Lactobacillus plantarum* BSGP201683, enterotoxigenic *Escherichia coli* K88, immune response, intestinal barrier, gut microflora

## Abstract

In this work, we searched for an effective probiotic that can help control intestinal infection, particularly enterotoxigenic *Escherichia coli* K88 (ETEC) invasion, in giant panda (*Ailuropoda melanoleuca*). As a potential probiotic strain, *Lactobacillus plantarum* BSGP201683 (*L. plantarum* G83) was isolated from the feces of giant panda and proven beneficial *in vitro*. This study was aimed to evaluate the protective effect of *L. plantarum* G83 in mice challenged with ETEC. The mice were orally administered with 0.2 mL of PBS containing *L. plantarum* G83 at 0 colony-forming units (cfu) mL^−1^ (control; negative control, ETEC group), 5.0 × 10^8^ cfu mL^−1^ (LDLP), 5.0 × 10^9^ cfu mL^−1^ (MDLP), and 5.0 × 10^10^ cfu mL^−1^ (HDLP) for 14 consecutive days. At day 15, the mice (LDLP, MDLP, HDLP, and ETEC groups) were challenged with ETEC and assessed at 0, 24, and 144 h. Animal health status; chemical and biological intestinal barriers; and body weight were measured. Results showed that *L. plantarum* G83 supplementation protected the mouse gut mainly by attenuating inflammation and improving the gut microflora. Most indices significantly changed at 24 h after challenge compared to those at 0 and 144 h. All treatment groups showed inhibited plasma diamine oxidase activity and _D_-lactate concentration. Tight-junction protein expression was down-regulated, and interleukin (IL)-1β, IL-6, IL-8, TLR4, and MyD88 levels were up-regulated in the jejunum in the LDLP and MDLP groups. The number of the *Enterobacteriaceae* family and the heat-labile enterotoxin (LT) gene decreased (*P* < 0.05) in the colons in the LDLP and MDLP groups. All data indicated that *L. plantarum* G83 could attenuate acute intestinal inflammation caused by ETEC infection, and the low and intermediate doses were superior to the high dose. These findings suggested that *L. plantarum* G83 may serve as a protective probiotic for intestinal disease and merits further investigation.

## Introduction

The giant panda is known as a rare, vulnerable species that is extremely popular worldwide. As a special herbivore, the giant panda has retained a typical carnivorous digestive system, which is easily afflicted by various intestinal diseases, especially ETEC infection which affects the intestinal barrier when the feed structure changes (Nataro and Kaper, 1998; Chen et al., 2015). Controlling this pathogen and its associated diarrhea heavily relies on antibiotics, of which their disadvantage could also threaten the animals. Probiotics are considered as one of the most effective alternatives to antibiotics because of their abilities to maintain or restore the normal microbiota, inhibit pathogen adhesion to intestinal walls, prevent inflammation, and protect intestinal barrier function (Geier et al., 2010; Archambaud et al., 2012; Yu et al., 2015).

Few reports are available on the role of probiotic strains in preventing ETEC invasion in giant panda. In a previous work, we isolated *L. plantarum* G83, a novel probiotic strain, from the feces of healthy captive giant panda. The microbe survived well at low pH, was tolerant to high bile-salt concentrations and resistant to antibiotics, and antagonized pathogenic bacteria *in vitro* (data not shown). For the next research step, the safety of this strain was assessed *in vivo*. Our laboratory also analyzed the intestinal microfloral structure of captive giant pandas at different age (Peng et al., 2016). The above-mentioned results served as the foundation for using the *L. plantarum* G83 as an antibiotic alternative and motivated the present study in mice. Similar to giant pandas, mice are omnivorous and easily infected by ETEC. Thus, mice are adopted as models for the study of the protective effects of *L. plantarum* G83 against ETEC invasion in giant panda.

## Materials and methods

### Bacterial strains and preparation

*L. plantarum* G83 was grown in De Man, Rogosa, and Sharp broth at 37°C for 24 h. Meanwhile, the ETEC K88 strain (O8:H19:F4ac^+^, LT^+^, STa^−^, STb^+^) was obtained from the China Institute of Veterinary Drug Control (Beijing, China) and grown in Luria–Bertani broth containing 5% fetal bovine serum at 37°C for 12 h.

The *L. plantarum* G83 cells were harvested by centrifugation at 3,000 × g for 10 min at 4°C after growing in an anaerobic environment at 37°C for 24 h. The cell pellet was washed with sterile physiological saline twice. Finally, the cells were diluted to 5.0 × 10^8^, 5.0 × 10^9^, and 5.0 × 10^10^ colony-forming units (cfu) mL^−1^ in 200 μL of PBS for 14 consecutive days.

Meanwhile, the ETEC cells were harvested by centrifugation at 3,000 × g for 10 min at 4°C after being incubated at 37°C for 12 h with vigorous shaking. Next, the cell pellet was washed with sterile physiological saline twice. The cells were then diluted to 1 × 10^9^ cfu mL^−1^ in PBS for the infectious challenge.

### Animals and experimental design

Ninety male ICR mice (16 ± 2 g average weight) were purchased from Chengdu Dashuo Biological Institute (Chengdu, China) and fed with commercial chow. All mice were housed in a temperature- and humidity-controlled room with a 12 h light/dark cycle and allowed free access to food and water.

The animals were randomly divided into five groups of 18 mice each. Each group was given different treatments as follows: (1) oral administration of sterile PBS from day 1 to day 15 (CONT), (2) oral administration of sterile PBS from day 1 to day 14 followed by oral challenge with ETEC on day 15 (ETEC), (3) oral administration of low-dose *L. plantarum* G83 (5.0 × 10^8^ cfu mL^−1^) from day 1 to day 14 followed by oral challenge with ETEC on day 15 (LDLP), (4) oral administration of intermediate-dose *L. plantarum* G83 (5.0 × 10^9^ cfu mL^−1^) from day 1 to day 14 followed by oral challenge with ETEC on day 15 (MDLP), and (5) oral administration of high-dose *L. plantarum* G83 (5.0 × 10^10^ cfu mL^−1^) on day 1 to day 14 followed by oral challenge with ETEC on day 15 (HDLP). Simultaneously, individual body weights were recorded daily.

After blood sampling, the animals were killed by cervical dislocation. All animal experiments were performed in accordance with the guidelines for the care and use of laboratory animals approved by the Institutional Animal Care and Use Committee of Sichuan Agricultural University (No. SYXKchuan 2014-187).

### Blood sample analysis

At 0, 24, and 144 h after ETEC challenge, blood samples were collected from the orbital venous plexus. The leukocyte level and population distribution were detected through a PE-6800 VET Fully Auto-hematology Analyzer.

At 0, 24, and 144 h after ETEC challenge, the blood samples were centrifuged at 2,000 × g for 20 min at 4°C. The resultant sera were obtained and stored at −80°C. The levels of immunoglobulin A (IgA), immunoglobulin M (IgM), immunoglobulin G (IgG), _D_-lactate, and diamine oxidase (DAO) activity were then analyzed by using ELISA kits (Shanghai MLBIO Biotechnology Co. Ltd., China).

### Relative quantitative real-time PCR

Total RNA was extracted from liquid-nitrogen-frozen jejuna using RNAiso plus (TaKaRa, Dalian, China) in accordance with the manufacturer's instructions. Meanwhile, RNA integrity and purity were assessed using 1% agarose gel electrophoresis and Nano Drop spectrophotometry (Nano Drop Technologies, Wilmington, DE, USA), respectively. Then, 1 μg of the total RNA was reverse transcribed using the PrimeScript® RT reagent Kit with gDNAEraser (TaKaRa, China) in compliance with the manufacturer's protocol. The generated cDNA was stored at −80°C until real-time PCR analysis.

All the genes detected and specific primers used are listed in Table 1. Quantitative real-time PCR was performed using a CFX Connect™ Real-time PCR Detection System (Bio-Rad, Hercules, CA, USA) with a SYBR Premix Ex Taq™ II PCR kit (TaKaRa, China). The PCR conditions were as follows: 95°C for 1 min, 40 cycles of denaturation at 95°C for 15 s, annealing at 60°C for 30 s, and extension at 72°C for 30 s. Melting curve analyses were performed to monitor the purity of the PCR product. All reactions were run in triplicates. Relative gene expression levels were evaluated through the 2^−ΔΔC*t*^ method, where ΔΔC*t* = (C_t, target_ 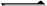 C_*t*, β−actin_)_treated group_ 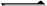 (C_*t*, target_ 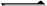 C_*t*, β−actin_)_control group_.

**Table 1:** Primers used for the relative quantification.

**Genes**	**Primer sequence (5′ → 3′)**	**Annealing temp (°C)**	**References**
β-actin	F:GTCCACCTTCCAGCAGATGT	60	Ren et al., 2013
	R:GAAAGGGTGTAAAACGCAGC		
IL-1β	F:ATGAAAGACGGCACACCCAC	60	Ren et al., 2013
	R:GCTTGTGCTCTGCTTGTGAG		
IL-6	F:TGCAAGAGACTTCCATCCAGT	60	Ren et al., 2013
	R:GTGAAGTAGGGAAGGCCG		
IL-8	F:CGGCAATGAAGCTTCTGTAT	60	Liu et al., 2015
	R:CCTTGAAACTCTTTGCCTCA		
IL-10	F:GACAACATACTGCTAACCGACTC	60	Yin et al., 2009
	R:ATCACTCTTCACCTGCTCCACT		
TLR2	F:GAATTGCATCACCGGTCAGAA	60	Ren et al., 2013
	R:CCTCTGAGATTTGACGCTTTGTC		
TLR4	F:TTCAGAACTTCAGTGGCTGGATT	60	Ren et al., 2013
	R:CCATGCCTTGTCTTCAATTGTTT		
MyD88	F:GCATGGTGGTGGTTGTTTCTG	60	Ren et al., 2013
	R:GAATCAGTCGCTTCTGTTGG		
MCP-1	F:GGTCCCTGTCATGCTTCTGG	60	Yin et al., 2009
	R:CCTGCTGCTGGTGATCCTCT		
Claudin-1	F:GGGGACAACATCGTGACCG	60	Zhong et al., 2010
	R:AGGAGTCGAAGACTTTGCACT		
Occludin	F:TTGAAAGTCCACCTCCTTACAGA	60	Zhong et al., 2010
	R:CCGGATAAAAAGAGTACGCTGG		
ZO-1	F:GATCCCTGTAAGTCACCCAGA	60	Zhong et al., 2010
	R:CTCCCTGCTTGCACTCCTATC		

*IL-1β, interleukin-1 beta; IL-6, interleukin-6; IL-8, interleukin-8; IL-10, interleukin-10; TLR2, toll like receptor 2; TLR4, toll like receptor 4; MyD88, myeloid differentiation primary response gene 88; MCP-1, monocyte chemotactic protein 1, ZO-1, zoluna occludens protein 1*.

### Real-time PCR quantification for colon microflora

The sample of total DNA for colon microflora (*n* = 6, each group, randomly) was extracted by using the E.Z.N.A.® Stool DNA kit (Omega Biotechnology, USA). The DNA concentration was detected by a Nano Drop spectrophotometer (Nano Drop Technologies, Wilmington, DE, USA). The primers, annealing temperatures, and product sizes for the different bacterial and total bacterial quantifications were displayed in Table 2. The PCR conditions were as same as those stated under *Relative Quantitative Real-time PCR* but with different annealing temperatures. The same approach (Rinttilä et al., 2004) was also used to quantify the LT.

**Table 2:** Primers used for the absolute quantification of bacterial 16S rDNA copy numbers.

**Target species**	**Primer sequence (5′ → 3′)**	**Annealing temp (°C)**	**Product size (bp)**	**References**
Total bacteria	F:ACTCCTACGGGAGGCAGCAG	59.5	200	Guo et al., 2008
	R:ATTACCGCGGCTGCTGG			
*Firmicutes*	F:GGAGYATGTGGTTTAATTCGAAGCA	60	126	Guo et al., 2008
	R:AGCTGACGACAACCATGCAC			
*Bacteroidetes*	F:GGARCATGTGGTTTAATTCGATGAT	58	126	Guo et al., 2008
	R:AGCTGACGACAACCATGCAG			
*Lactobacillus* group	F:AGCAGTAGGGAATCTTCCA	55	341	Rinttilä et al., 2004
	R:CACCGCTACACATGGAG			
*Bifidobacterium* spp.	F:TCGCGTC(C/T)GGTGTGAAAG	62	243	Rinttilä et al., 2004
	R:CCACATCCAGC(A/G)TCCAC			
*Streptococcus* spp.	F: AGAGTTTGATCCTCCGTCAG	58	144	Fiesel et al., 2014
	R:GTTAGCCGTCCCTTTCTGG			
*Enterococcus* spp.	F:CCCTTATTGTTAGTTGCCATCATT	52	144	Rinttilä et al., 2004
	R:ACTCGTTGTACTTCCCATTGT			
*Enterobacteriaceae* family	F:CATTGACGTTACCCGCAGAAGAAGC	52	195	Bartosch et al., 2004
	R:CTCTACGAGACTCAAGCTTGC			
LT	F:TCTCTATGTGCATACGGAGC	60	322	Barletta et al., 2013
	R:CCATACTGATTGCCGCAAT			

### Statistical analysis

All data were expressed as means and standard deviations. Statistical analysis was performed by one-way ANOVA in SPSS 19.0. Differences among treatments were compared using the Student-Newman-Keuls multiple comparison test. Statistical significance was set at *P* ≤ 0.05.

## Results

### Clinical symptoms and growth performance

**Figure 1:**
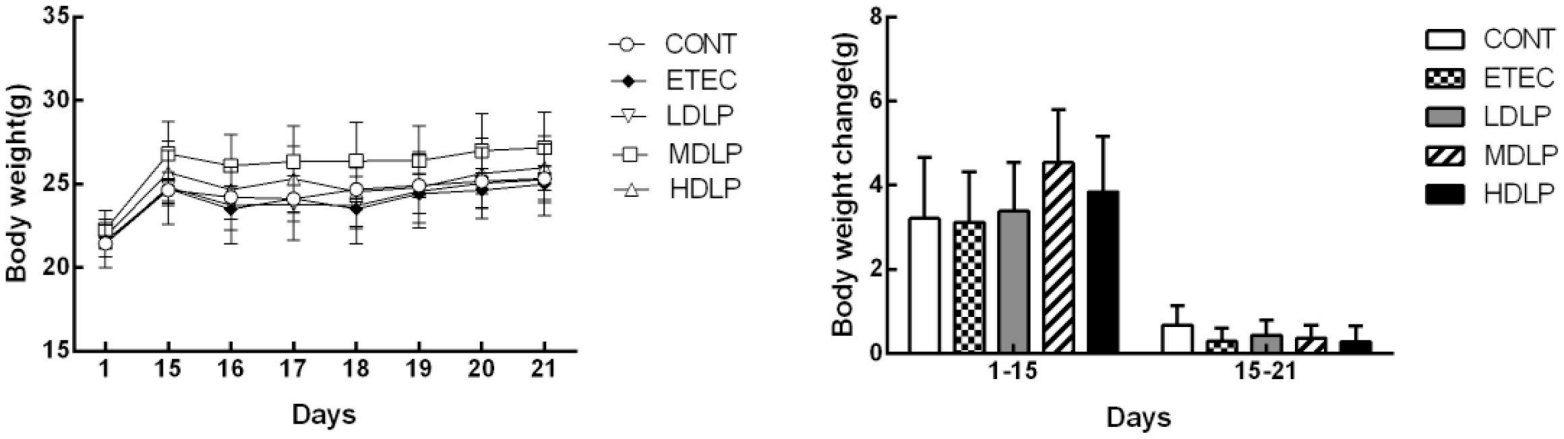
Dose effect of *Lactobacillus plantarum* on the body weight of mice during pre- and post-challenge periods (*n* = 6). CONT, control group, treatment with PBS at all period; ETEC, negative group, oral challenge with ETEC on day 15; LDLP, low dose (5.0 × 10^8^ cfu mL^−1^) group; MDLP, intermediate dose (5.0 × 10^9^ cfu mL^−1^) group; HDLP, high dose (5.0 × 10^10^ cfu mL^−1^) group; LDLP, MDLP, and HDLP groups were infected with ETEC at day 15. There was no significant (*P* > 0.05) in all groups.

Before the ETEC challenge, no clinical sign was observed in the mice of any experimental group. After the ETEC challenge, only one mouse from the ETEC group presented with mild diarrhea, whereas most of the mice initially became dull and depressed and then lost weight. All groups showed no significant difference (*P* > 0.05) in body weight gain throughout the entire trial period (Figure 1). However, the mice given *L. plantarum* gained more body weight than those by others. After ETEC challenge, the ETEC, LDLP, MDLP, and HDLP groups presented similar weight changes (Figure 1).

### Leukocyte analysis

**Figure 2:**
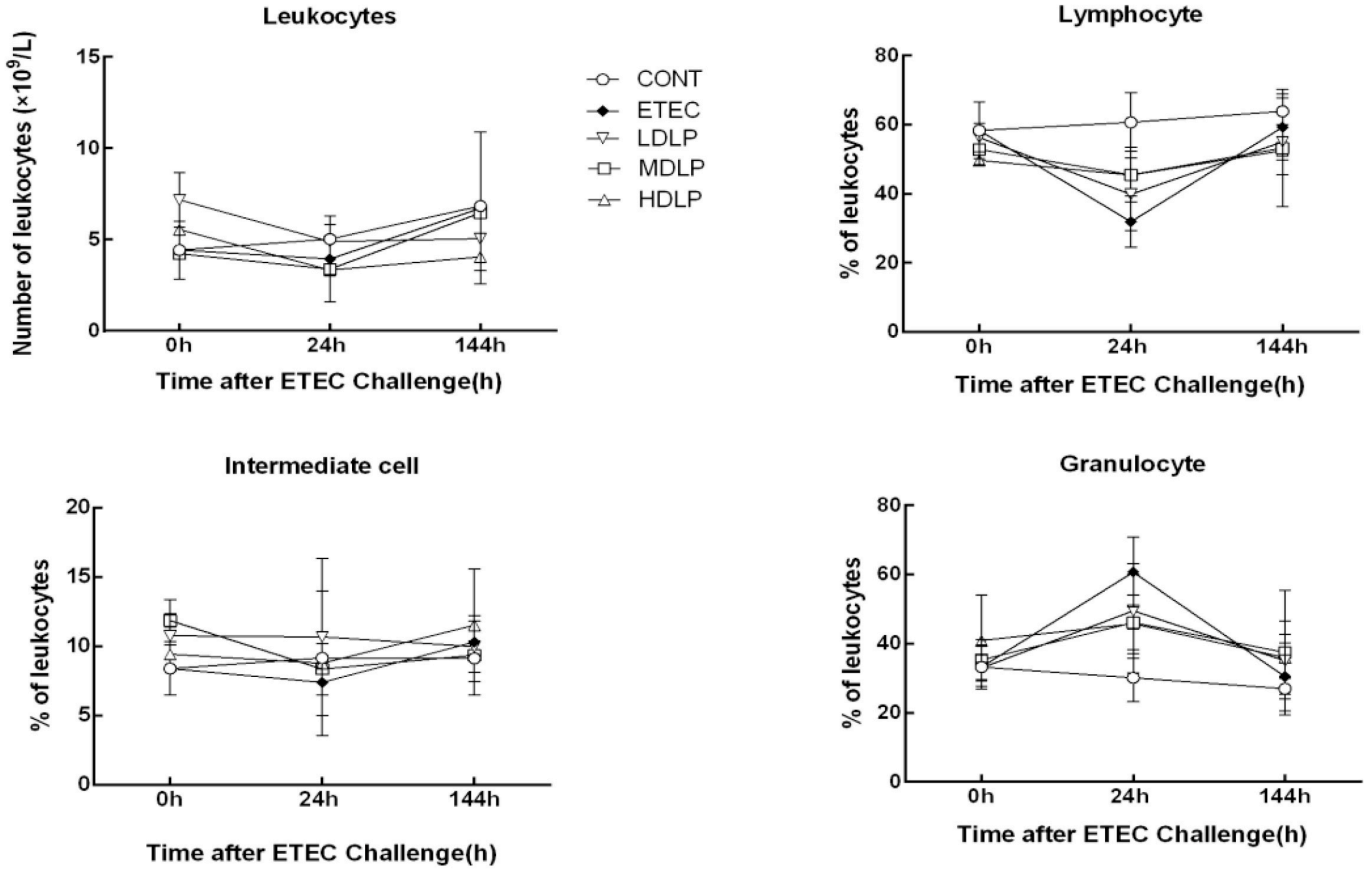
Dose effect of *Lactobacillus plantarum* on blood leukocyte count and population distribution of mice during pre- and post-challenge periods. CONT, control group, treatment with PBS at all period; ETEC, negative group, oral challenge with ETEC on day 15; LDLP, low dose (5.0 × 10^8^ cfu mL^−1^) group; MDLP, intermediate dose (5.0 × 10^9^ cfu mL^−1^) group; HDLP, high dose (5.0 × 10^10^ cfu mL^−1^) group; LDLP, MDLP, and HDLP groups were infected with ETEC at day 15. At 24 h after ETEC challenge, lymphocyte concentrations decreased (*P* < 0.05) and granulocyte concentrations increased (*P* < 0.05) in ETEC group and *Lactobacillus*-supplemented groups as compared to CONT group.

Figure 2 shows the changes in the number of leukocytes, lymphocytes, intermediate cells (including monocyte, eosinophils, and basophils), and granulocytes numbers in the five groups after ETEC challenge. At 24 h after ETEC challenge, the lymphocyte concentrations decreased (*P* < 0.05) and the granulocyte concentrations increased (*P* < 0.05) in the ETEC group and *Lactobacillus*-supplemented groups relative to those in the CONT group. No significant difference (*P* > 0.05) was noted among the ETEC group and the *Lactobacillus*-supplemented groups. At 0, 24, and 144 h after ETEC challenge, the levels of leukocyte, lymphocyte, intermediate cell, and granulocyte did not change significantly (*P* > 0.05) in all groups.

### Serum DAO activity and _D_-lactate concentration

**Figure 3:**
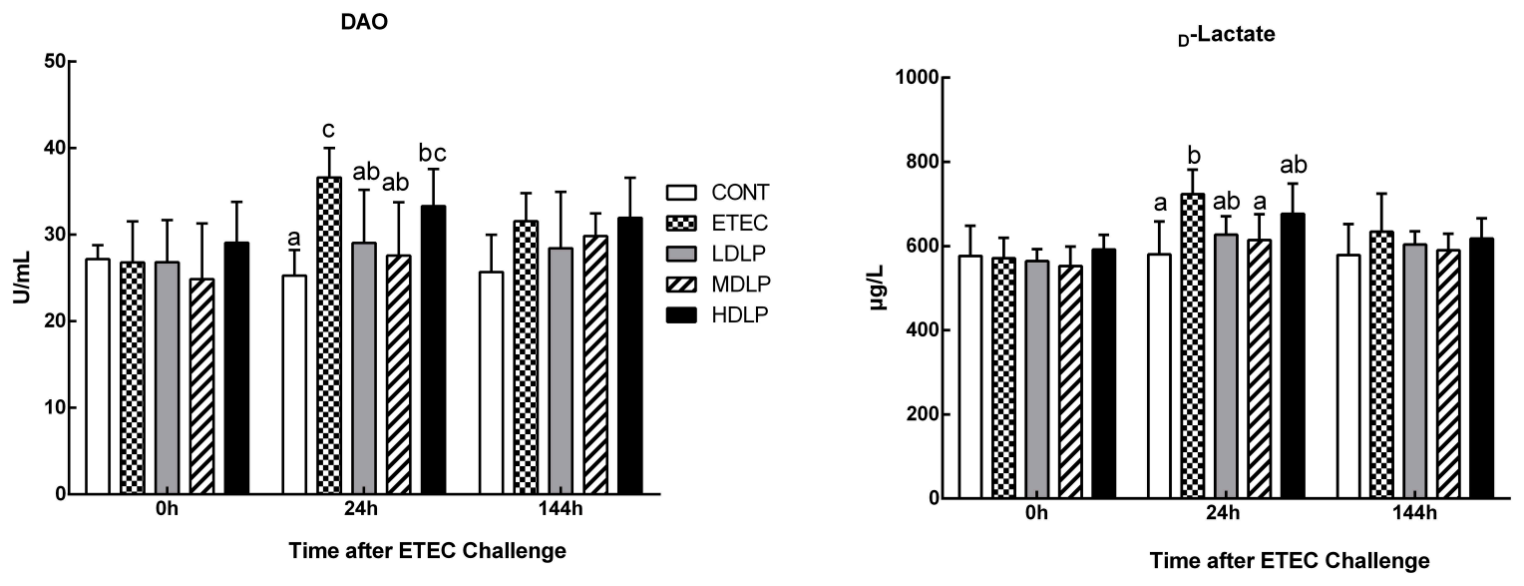
Dose effect of *Lactobacillus plantarum* on DAO activity and _D_-lactate concentration in serum of mice during pre- and post-challenge periods. CONT, control group, treatment with PBS at all period; ETEC, negative group, oral challenge with ETEC on day 15; LDLP, low dose (5.0 × 10^8^ cfu mL^−1^) group; MDLP, intermediate dose (5.0 × 10^9^ cfu mL^−1^) group; HDLP, high dose (5.0 × 10^10^ cfu mL^−1^) group; LDLP, MDLP, and HDLP groups were infected with ETEC at day 15. Bars with different letters are significantly different (*P* < 0.05). Bars share the same letters do not differ significantly (*P* > 0.05).

The levels of DAO and _D_-lactate after ETEC challenge are shown in Figure 3. At 24 h after ETEC challenge, the DAO and _D_-lactate concentrations in the ETEC group were significantly higher (*P* < 0.05) than those in the CONT and MDLP groups but did not differ (*P* > 0.05) from those of the HDLP group. At 0 and 144 h after ETEC challenge, no difference (*P* > 0.05) in DAO and _D_-lactate concentrations was noted in all the groups.

### Effects of *L. plantarum* on the serum concentrations of IgG, IgA, and IgM

**Figure 4:**
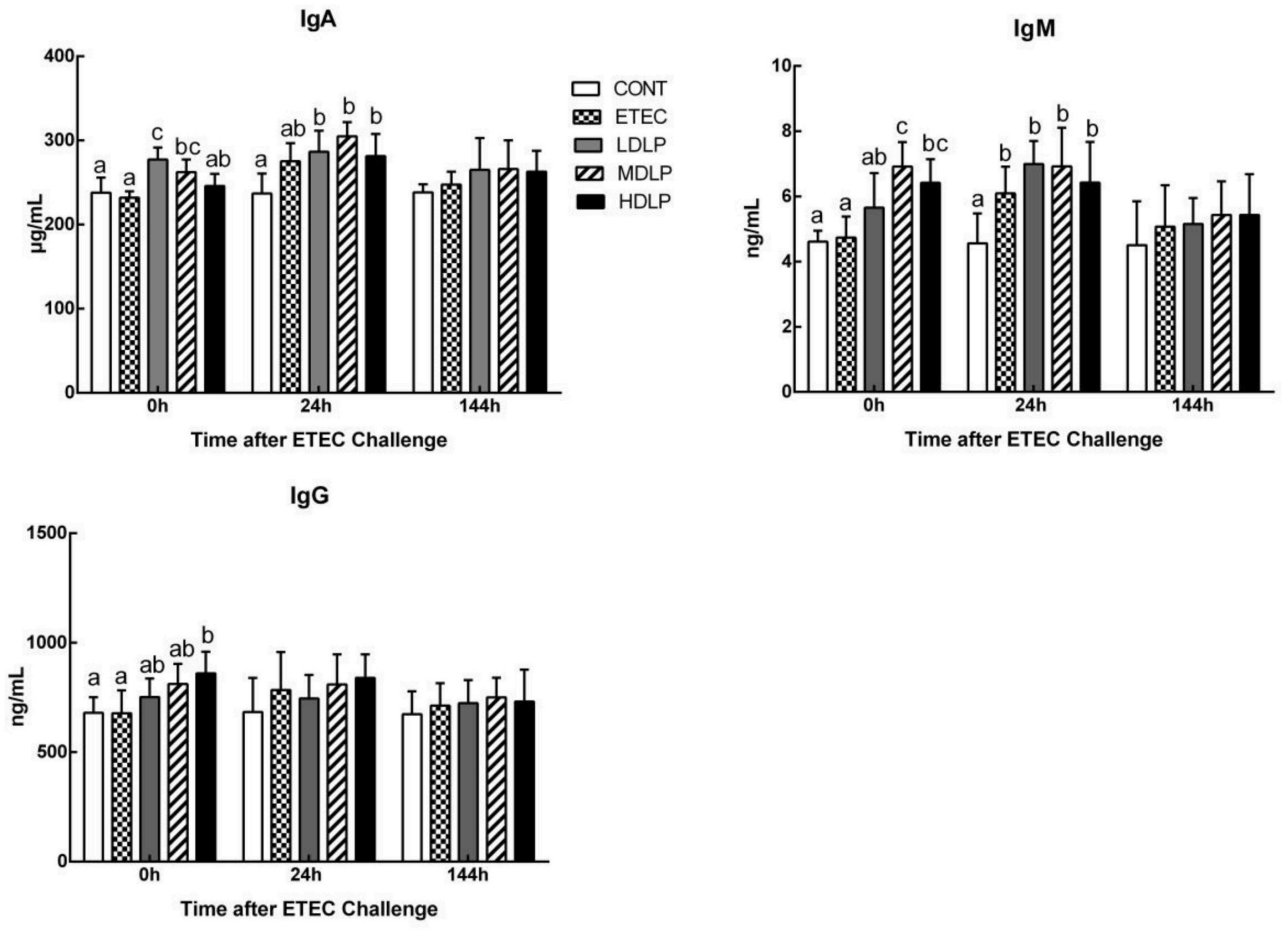
Dose effect of *Lactobacillus plantarum* on concentrations of IgA, IgM and IgG in serum of mice during pre- and post-challenge periods. CONT, control group, treatment with PBS at all period; ETEC, negative group, oral challenge with ETEC on day 15; LDLP, low dose (5.0 × 10^8^ cfu mL^−1^) group; MDLP, intermediate dose (5.0 × 10^9^ cfu mL^−1^) group; HDLP, high dose (5.0 × 10^10^ cfu mL^−1^) group; LDLP, MDLP, and HDLP groups were infected with ETEC at day 15. Bars with different letters are significantly different (*P* < 0.05). Bars share the same letters do not differ significantly (*P* > 0.05).

Before challenge, the serum IgA levels in the LDLP and MDLP groups significantly increased (*P* < 0.05) with respect to that in the CONT group. Meanwhile, the IgM concentrations in the MDLP and HDLP groups were higher (*P* < 0.05) than that in the CONT. However, only the IgG level in HDLP increased (*P* < 0.05) with respect to that in the CONT. At 24 h after ETEC challenge, the IgA and IgM contents in the LDLP, MDLP, and HDLP groups were higher (*P* < 0.05) than those in the CONT. At 144 h post-challenge, no significant difference in IgA, IgM, or IgG level (*P* > 0.05) was observed among the groups (Figure 4).

### Effect of *L. plantarum* on the expression of tight junction (TJ)-associated proteins in the jejunum

**Figure 5:**
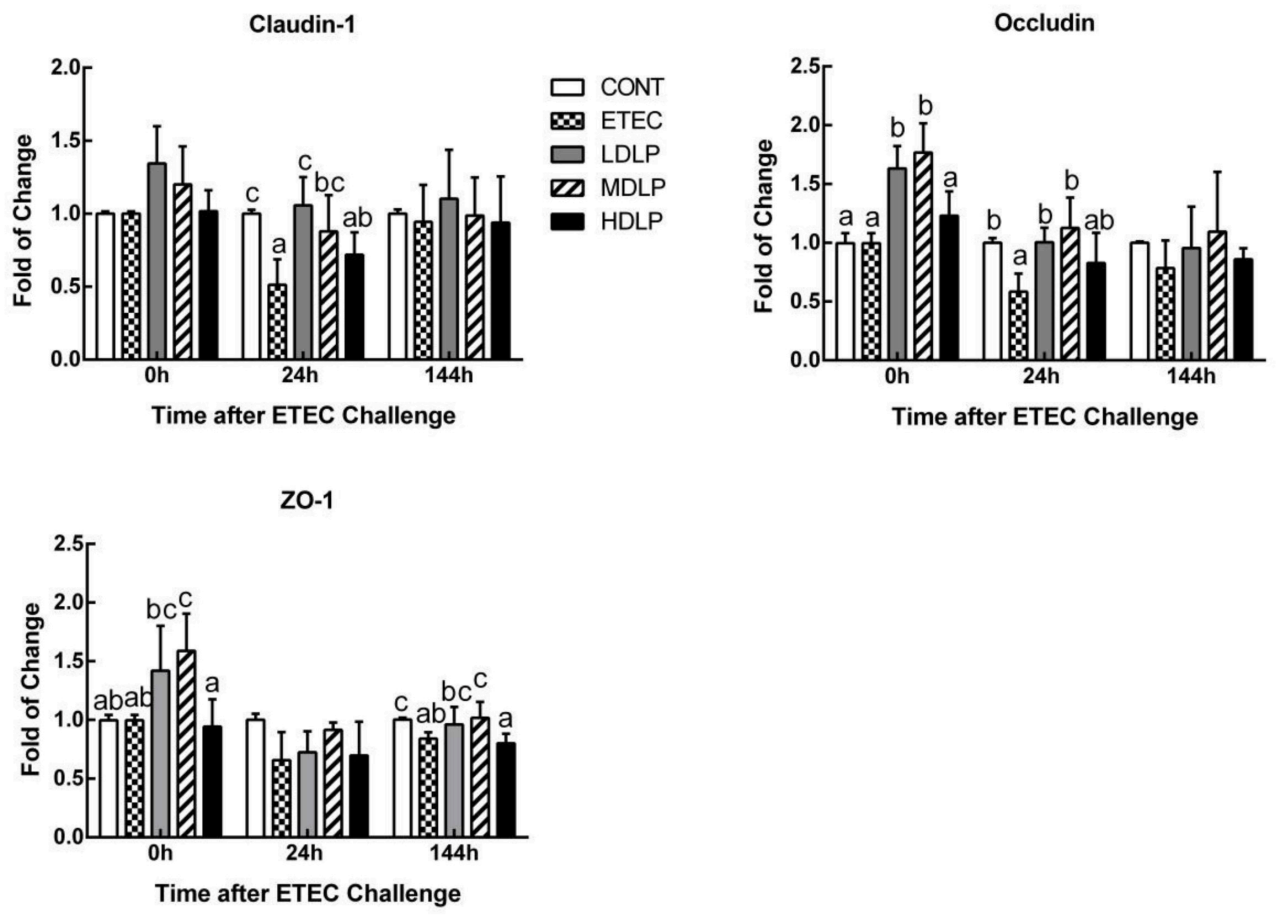
Dose effect of *Lactobacillus plantarum* on tight junction proteins mRNA expression in jejunum of mice during pre- and post-challenge periods. CONT, control group, treatment with PBS at all period; ETEC, negative group, oral challenge with ETEC on day 15; LDLP, low dose (5.0 × 10^8^ cfu mL^−1^) group; MDLP, intermediate dose (5.0 × 10^9^ cfu mL^−1^) group; HDLP, high dose (5.0 × 10^10^ cfu mL^−1^) group; LDLP, MDLP, and HDLP groups were infected with ETEC at day 15. Bars with different letters are significantly different (*P* < 0.05). Bars share the same letters do not differ significantly (*P* > 0.05).

Before challenge, the MDLP group exhibited an up-regulated expression (*P* < 0.05) of occludin and zonula occluden 1 (ZO-1), whereas the LDLP group showed only an increased expression (*P* < 0.05) of occludin, with respect to those in the CONT group. At 24 h after ETEC challenge, the expression of all the TJ-associated proteins was down-regulated, and the ETEC group achieved lower claudin-1 and occludin levels than those in the CONT group (*P* < 0.05). However, the difference among the CONT, LDLP, and MDLP groups was not significant (*P* > 0.05). At 144 h after challenge, the expression of claudin-1 and occludin in all groups was not significant (*P* > 0.05). The expression of ZO-1 in ETEC and HDLP groups was down-regulated (*P* < 0.05) compared to CONT group (Figure 5).

### Effect of *L. plantarum* on the expression of cytokines and toll-like receptors (TLRs) in the jejunum

**Figure 6:**
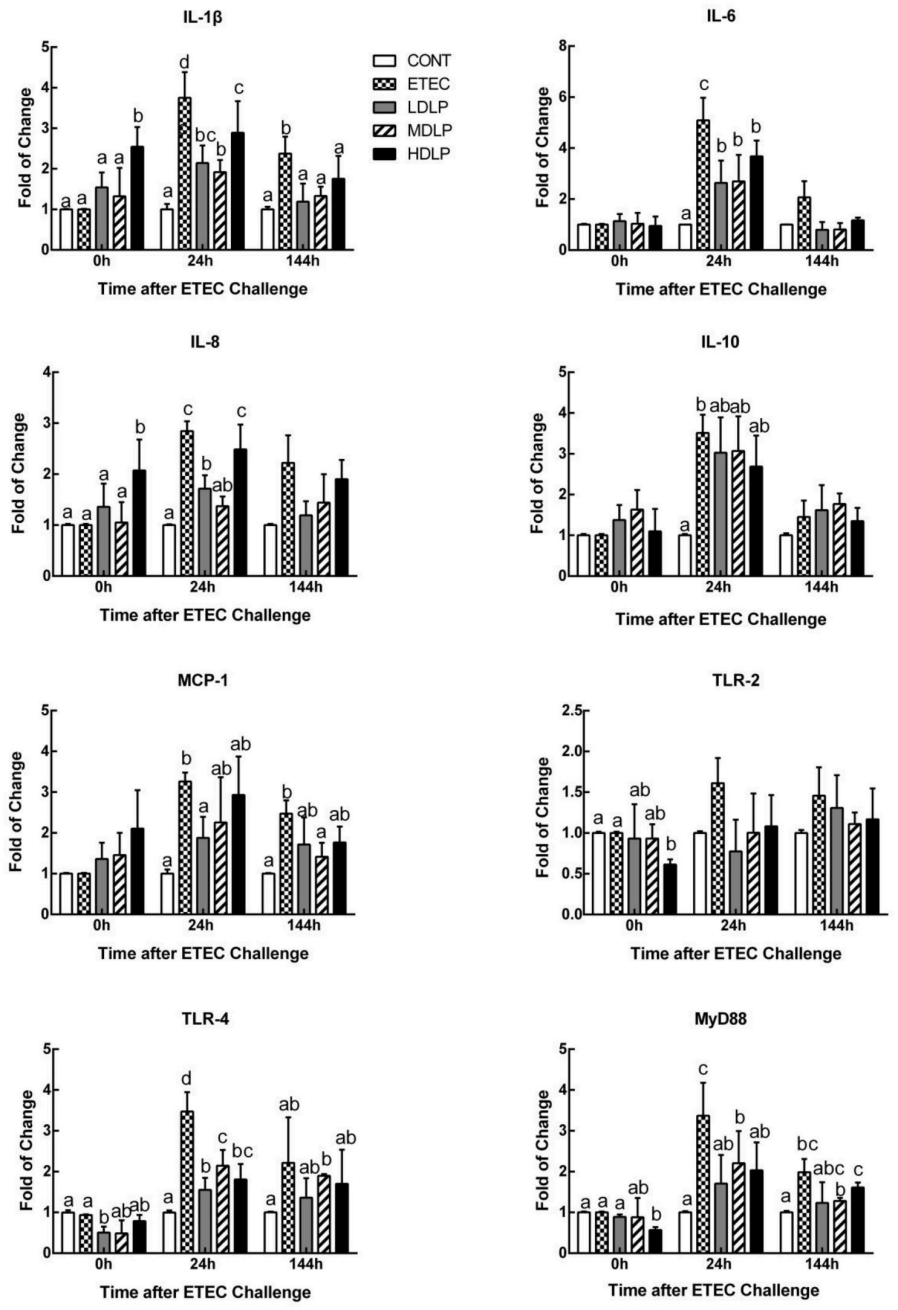
Dose effect of *Lactobacillus plantarum* on cytokines and Toll-like receptor mRNA expression in jejunum of mice during pre- and post-challenge periods. CONT, control group, treatment with PBS at all period; ETEC, negative group, oral challenge with ETEC on day 15; LDLP, low dose (5.0 × 10^8^ cfu mL^−1^) group; MDLP, intermediate dose (5.0 × 10^9^ cfu mL^−1^) group; HDLP, high dose (5.0 × 10^10^ cfu mL^−1^) group; LDLP, MDLP, and HDLP groups were infected with ETEC at day 15. Bars with different letters are significantly different (*P* < 0.05). Bars share the same letters do not differ significantly (*P* > 0.05).

Interleukin (IL)-1β and IL-8 levels in the HDLP group obviously rose (*P* < 0.05) relative to those of the CONT group at 0 h after ETEC challenge. At 24 h after ETEC challenge, the IL-1β, IL-8, IL-6, TLR4, and MyD88 levels in the ETEC group were significantly higher (*P* < 0.05) than those of the CONT, LDLP, and MDLP groups. Meanwhile, the IL-8 contents did not significantly change (*P* > 0.05) between the ETEC and HDLP groups. At 144 h, the IL-1β expression in the ETEC group was higher (*P* < 0.05) than those of the other groups (Figure 6).

### Microbial analysis in the colon

**Figure 7:**
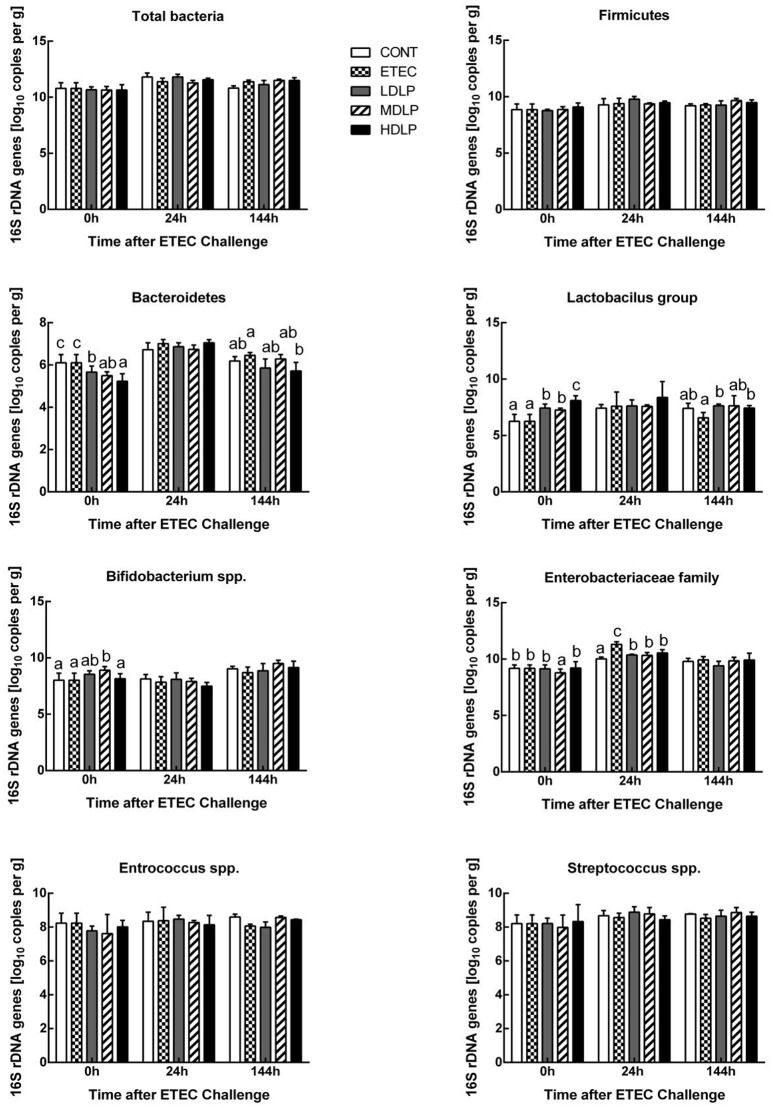
Dose effect of *Lactobacillus plantarum* on composition of the mice colonic microbiota during pre- and post-challenge periods. CONT, control group, treatment with PBS at all period; ETEC, negative group, oral challenge with ETEC on day 15; LDLP, low dose (5.0 × 10^8^ cfu mL^−1^) group; MDLP, intermediate dose (5.0 × 10^9^ cfu mL^−1^) group; HDLP, high dose (5.0 × 10^10^ cfu mL^−1^) group; LDLP, MDLP, and HDLP groups were infected with ETEC at day 15. Bars with different letters are significantly different (*P* < 0.05). Bars share the same letters do not differ significantly (*P* > 0.05).

At 0 h, the *Lactobacillus* concentration of the HDLP group and the *Bifidobacterium* spp. content of the MDLP group was significantly higher (*P* < 0.05) compared to the CONT group. Meanwhile, the *Bacteroidetes* level in the LDLP, MDLP, and HDLP group and the *Enterobacteriaceae* level in the MDLP decreased (*P* < 0.05). At 24 h, the *Enterobacteriaceae* concentration in the ETEC group grew (*P* < 0.05) relative to those of the other groups. At 144 h, all bacterial concentrations apart from *Bacteroidetes* and *Lactobacillus* did not considerably vary among the groups (Figure 7).

### Effect of *L. plantarum* on the LT gene levels in the colon

**Figure 8:**
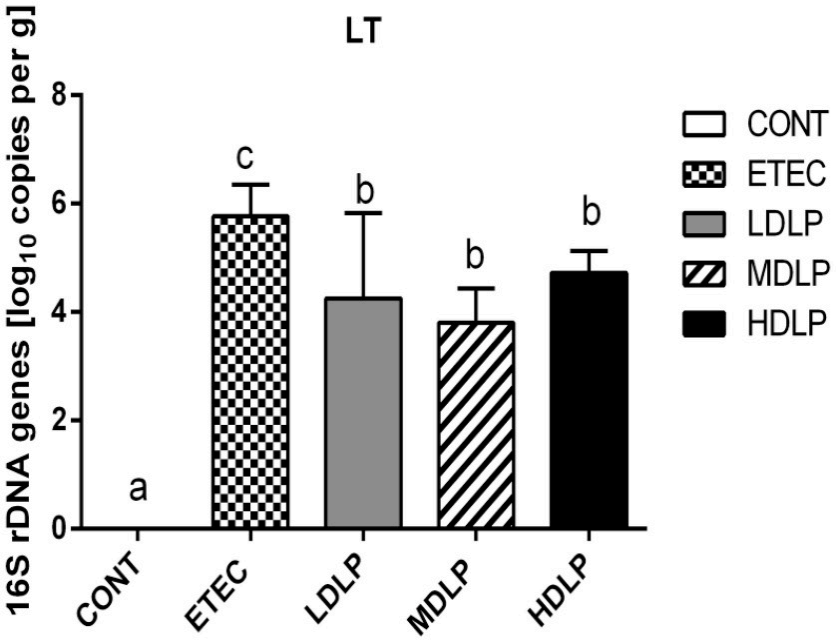
Amount of LT gene in colon of mice at 24 h after ETEC challenge. CONT, control group, treatment with PBS at all period; ETEC, negative group, oral challenge with ETEC on day 15; LDLP, low dose (5.0 × 10^8^ cfu mL^−1^) group; MDLP, intermediate dose (5.0 × 10^9^ cfu mL^−1^) group; HDLP, high dose (5.0 × 10^10^ cfu mL^−1^) group; LDLP, MDLP, and HDLP groups were infected with ETEC at day 15. Bars with different letters are significantly different (*P* < 0.05). Bars share the same letters do not differ significantly (*P* > 0.05).

The LT levels in the colon of the ETEC group were significantly higher (*P* < 0.05) than those of the other groups. Moreover, LT was not detected at 0 and 144 h after challenge (Figure 8).

## Discussion

Severe gut disease can damage host health. However, in giant panda, this kind of disease must be prevented and treated. The relationship between probiotics and improved gut health has received considerable scientific interest for more than a century. To protect animal guts, scientists have shown that probiotics, such as *Lactobacillus, Bifidobacterium*, and *Bacillus*, prevent gut bacterial disease (Barba-Vidal et al., 2017; Nishida et al., 2017; Wu et al., 2017). In the present study, *L. plantarum* G83 was isolated from the feces of healthy giant panda and confirmed beneficial *in vitro*. This current work aimed to evaluate the protective effect of *L. plantarum* G83 in mice challenged with ETEC. To accomplish this objective, we assessed mice for their weights, as well as chemical and biological intestinal barriers.

In the present study, the weights and gain ratios in the ETEC group were certainly lower than those of the other groups. However, the growth performance did not significantly change in the mice supplemented with *L. plantarum* G83 either before or after ETEC infection. This result was similar to those of previous works (Nguyen et al., 2007; Wang et al., 2009, 2016) but can otherwise be explained by the shortage of weight gain evaluation period for weight gain. After ETEC infection, the mice only showed weight loss and slow growth rather than the clear clinical signs of diarrhea and death. Such presentation may be due to the differences in sensitivity of the model animal and in virulence of the strain (Duchet-Suchaux et al., 1990; Porter et al., 2011). These results revealed that the strain did not obviously affect the gain weight within a short period.

Maintaining the intestinal epithelial barrier integrity is important for the defense against pathogen invasion and inflammation (Fasano and Shea-Donohue, 2005). In this regard, plasma DAO and _D_-lactate have been proposed as circulating markers for the extent of damage and repair of the intestinal mucosa (Wijtten et al., 2011; Zhao et al., 2014). These markers can reflect the intestinal permeability and barrier function in peripheral blood (Ewaschuk et al., 2005). Furthermore, TJs, such as claudin-1, occludin and ZO-1, have been proven to seal the lateral intercellular space and achieve an intact layer of epithelial cells that guard against pathogens (Groschwitz and Hogan, 2009; Suzuki, 2013). In the present study, the intermediate- and low-dose groups notably inhibited the increase in plasma DAO and _D_-lactate levels after ETEC infection. ETEC invasion has been reported to increase the release of DAO and _D_-lactate in the plasma and damage the intestinal epithelial cell membrane (Roselli et al., 2007; Yang et al., 2014; Xun et al., 2015). Hence, our results indicated that the strain exerts a protective effect on the host during ETEC invasion. Different doses of *L. plantarum* G83 prevented ETEC-induced membrane damage by inhibiting the delocalization of ZO-1 and consequently raising occludin and claudin-1 amounts. This effect is consistent with that observed by Roselli et al. (2007), who found higher occludin and ZO-1 mRNA expression in piglets fed with *Lactobacillus sobrius* than in those not fed with the strain. In the former piglets, ETEC-induced membrane damage was prevented by inhibiting the rearrangement of F-actin and dephosphorylation of occludin. As Mennigen and Bruewer (2009) reported, there is a close link between probiotics and regulation of intestinal permeability and barrier function. The above-mentioned findings collectively indicated that *L. plantarum* G83 exerts a protective effect against ETEC-induced membrane damage.

During infection, innate or acquired immunity is activated. Immunocytes then play a key role in the innate and adaptive immune responses. Immunocyte number and variation reflect the immunity or infection status (Demissie et al., 2013; Hunt et al., 2015). Furthermore, the immunocyte induction of cytokine and antibody production participates in immune regulation and the inflammatory response (Baehner, 1975; Cassatella, 1999). One signaling pathway that promotes the expression of pro-inflammatory genes is the TLR- and MyD88-dependent pathways (Zughaier et al., 2005). In the current study, the *L. plantarum* G83 strain did not significantly alter the leukocyte amount and variation in the mouse peripheral blood among the groups at different time points. Besides the granulocyte, the amount of leukocytes, lymphocytes, and intermediate cells decreased at 24 h after ETEC inoculation. This effect may have been caused by the high expression of IL-8 and MCP-1, which take part in the immunocytes migration to the site of infection in the intestine. Such results were similar to those of previous reports (Zhu et al., 2014). *Lactobacillus* supplementation enhanced serum IgG, IgM, and IgA levels and indicated that probiotics could stimulate systemic or mucosal antibody response (Frece et al., 2006; Mizumachi et al., 2009). In the present study, the *L. plantarum* G83 obviously increased the IgA, IgM, and IgG levels before or after ETEC challenge. IgA has an important role in the protection of mucosal surfaces against pathogens (Galdeano and Perdigon, 2004). The secretory IgA of intestinal mucosa will be detected in next study. Low and intermediate doses of the strain also decreased the mRNA levels of IL-1β, −6, and −8 in the jejunum, probably by modifying the immunocyte profile. Early studies confirmed that lactobacilli lower serum IL-6 concentrations after acute ETEC challenge in piglets (Zhang et al., 2010). The aforementioned observations suggested that inflammation was ameliorated in the LDLP and MDLP groups and were consistent with those of previous studies where probiotics exerted dose-dependent effects (Mileti et al., 2009). Lee et al. (2012) found that high-dose *L. plantarum* CJLP243 (10^8^–10^10^ cfu kg^−1^) most substantially improved the growth and health performance of weaning pigs, especially those with bacterium-induced acute bowel inflammation. A recent trial of piglets challenged with F4^+^ ETEC demonstrated that administering LGG (10^12^ cfu day^−1^) failed to prevent F4^+^ ETEC infection. This failure was probably due to the disruption of microbial and inflammatory responses by excessive probiotic levels (Li et al., 2012). Same probiotic in different doses have different modulation effect on T-cell immune response and mucosal IgA response (Wen et al., 2012, 2015). Thus, only intermediate- and low-dose *L. plantarum* G83 positively affects acute inflammatory bowel disease.

Besides promoting nutritional digestion, intestinal microflora is also involved in host immunity and pathology. Bacterial infections or enteritis, antibiotic treatment, and immunosuppression may alter the population, quantity, or habitat of gut microflora and may lead to the excessive growth of opportunistic pathogens as well as microbial dysbiosis (Bouhnik et al., 2004; Taur and Pamer, 2013). Numerous studies showed that probiotics can regulate and maintain the balance of intestinal flora by enhancing indigenous bacterial colonization and/or competitive exclusion to battle pathogens (Gareau et al., 2010). The present results revealed that administering *L. plantarum* G83 not only increased the abundance of *Lactobacillus* and *Bifidobacterium*, but also decreased the number of *Bacteroidetes* and *Enterobacteriaceae* in the colon. Our results were also similar to those of the reports where LGG and *Lactobacillus* Bar13 greatly reduced the colonization of pathogenic *E. coli* and *Salmonella* (Collado et al., 2007; Candela et al., 2008). Probiotics secrete some bacteriocin that can kill or inhibit pathogens (Nakamura et al., 2013; Pandey et al., 2013). Furthermore, *Lactobacillus* and *Bifidobacterium* can also create a biomembrane on intestinal mucosa to serve as a barrier against colonizing pathogen and improve gut immunity (Collado et al., 2008; Jones and Versalovic, 2009; Chen et al., 2014). Probiotics can also compete with harmful bacteria for nutrition (Elli et al., 2000; Hooper, 2009). Overall, our results demonstrated that *L. plantarum* G83 is beneficial to host health.

## Conclusion

The probiotic *L. plantarum* G83 had a positive effect against pathogens mainly by chemical barrier and biological barrier, not only increasing the abundance of *Lactobacillus* and *Bifidobacterium*, but modulating immune response. In addition, different dose of *L. plantarum* G83 had various effects in modulating immune response. The results of this research may suggest that there is an opportunity to develop a new probiotic product for use in giant panda. And more research is needed for fully guarantee the safety and efficacy of this strain.

## Author contributions

Designed the experiments: QL, XN, and ZP. Conceived and supervised the study: XN, QW, ZP, and LN. Performed the experiments: QL, YZ, and HS. Data analyzed by: QL, ZP, KP, and BJ. Wrote the manuscript: QL, ZP, and DZ. Proofread the manuscript: DZ and HW. All authors read and approved the final manuscript.

### Conflict of interest statement

The authors declare that the research was conducted in the absence of any commercial or financial relationships that could be construed as a potential conflict of interest.
